# MicroRNA-141 suppresses growth and metastatic potential of head and neck squamous cell carcinoma

**DOI:** 10.18632/aging.101791

**Published:** 2019-02-08

**Authors:** Zhiguo Zhao, Dan Gao, Tie Ma, Liping Zhang

**Affiliations:** 1 Department of Oral and Maxillofacial Surgery, Shengjing Hospital of China Medical University, Shenyang, P.R.China; 2 Department of Clinical Laboratory, Shengjing Hospital of China Medical University, Shenyang, P.R.China

**Keywords:** EGFR, HNSCC, miR-141, growth, metastasis

## Abstract

MicroRNAs (miRNAs) serve as regulatory factors in both healthy tissue and various cancers. Here, we used an miRNA microarray to screen for miRNAs differentially expressed between HNSCC and adjacent epithelial tissue. Among these, levels of miR-141 were significantly reduced in HNSCC tissues. Expression levels of epidermal growth factor receptor (EGFR) were enhanced in tissues with low miR-141 expression but were reduced by miR-141 overexpression, and there was a significant negative correlation between EGFR and miR-141 levels in HNSCC tissues (*P* < 0.01). Luciferase assays confirmed that miR-141 targeted EGFR mRNA. *In vitro*, miR-141 inhibited the proliferation and migration of Cal-27 and FaDu HNSCC cells with corresponding decreases in CDK4 and MMP2. miR-141 also enhanced the incidence of apoptosis among the cells with a corresponding decrease in bcl-2. In BALB/c mice injected with FaDu HNSCC cells, miR-141 mitigated hepatic metastasis and inhibited expression of EGFR, CDK4, bcl-2 and MMP2. These results suggest that miR-141 functions as a tumor suppressor in HNSCC and that it suppresses tumor growth and metastasis by suppressing EGFR signaling. MiR-141 thus appears to be a potentially useful therapeutic target in the treatment of HNSCC.

## Introduction

Head and neck squamous cell carcinoma (HNSCC) is the sixth most common type of human cancer, with more than 70% of patients suffering with recurrent or metastatic disease [1]. There is thus an urgent need for effective therapeutic regimens to treat HNSCC.

MiRNAs play fundamental roles in normal physiological and pathological processes, including cancer. miRNAs may affect the progression of cancer through their post-transcriptional regulation on genes [2]. For example, miR-141 is reportedly downregulated in several cancers, including hepatocellular carcinoma and gastric, colorectal, bladder and ovarian cancers [3–5]. However, the role of miR-141 in HNSCC remains unclear.

Epidermal growth factor receptor (EGFR) is a widely distributed cell surface receptor involved in the physiological processes of cell growth and differentiation as well as in cancer metastasis. More than 90% of HNSCCs exhibit upregulated EGFR expression [6,7]. Identification of key factors that inhibit EGFR expression may improve the treatment of HNSCC. In the present study, therefore, we examined the effects of miR-141 on EGFR expression in HNSCC cells. We also analyzed the effects of miR-141 on proliferation, apoptosis and migration of HNSCC cells *in vivo* and *in vitro*. Our findings suggest miR-141 acts as a tumor suppressor by targeting EGFR expression in HNSCC cells.

## RESULTS

### Identification of miRNAs differentially expression in HNSCC

**Figure 1:**
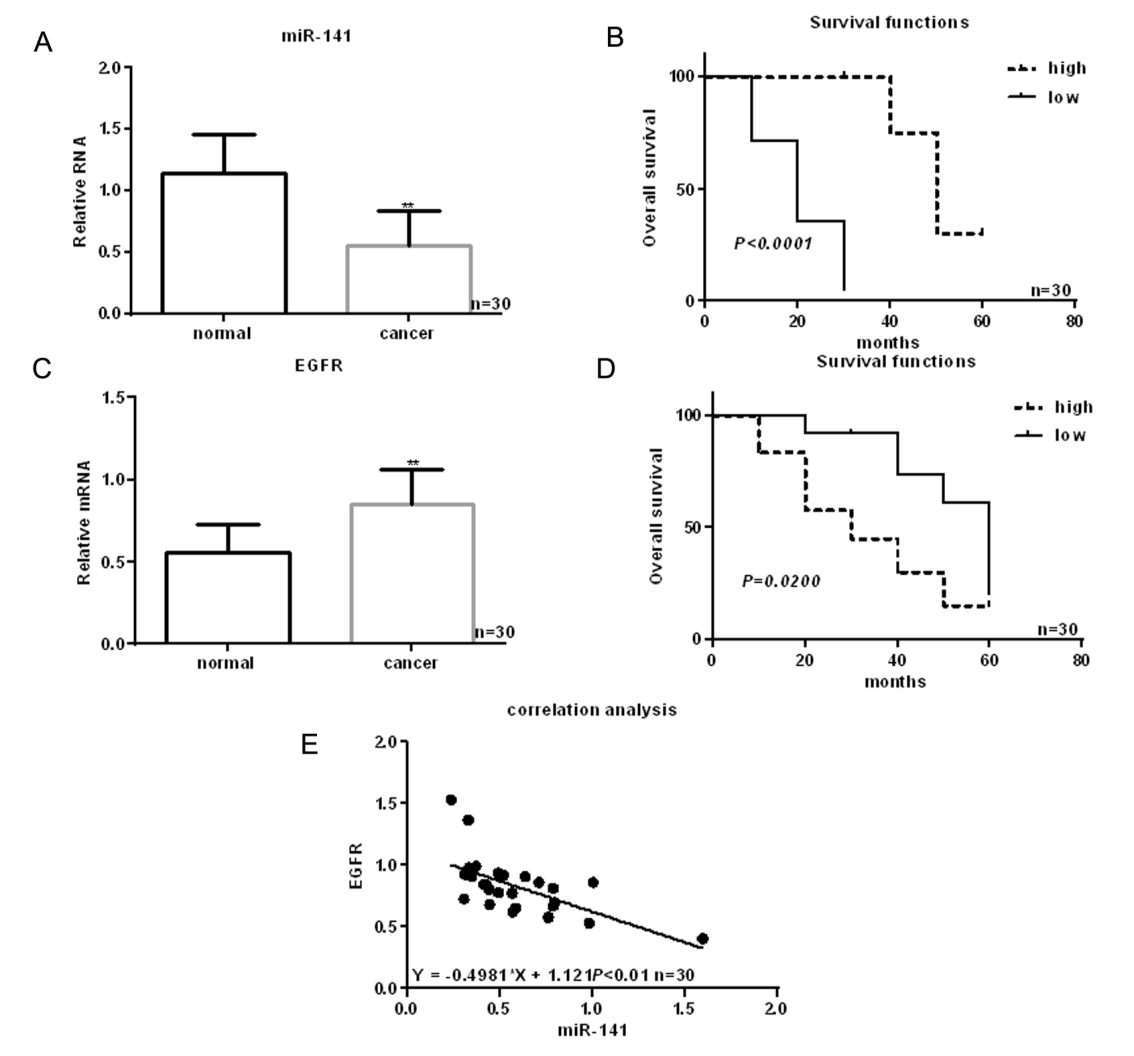
**Identification of miRNAs differentially expressed in HNSCC.** (**A**) Expression of miR-141 in HNSCC tissues and adjacent normal tissues detected using real time PCR. (**B**) Relationship between miR-141 and survival in HNSCC patients. (**C**) Expression of EGFR in HNSCC tissues and adjacent normal tissues detected using real time PCR. (**D**) Relationship between EGFR and survival in HNSCC patients. (**E**) Correlation between expression levels of miR-141 and EGFR in HNSCC. ** P< 0.05 vs. adjacent tissues.

A miRNA microarray was used to screen for abnormal expression of miRNAs in 30 samples of paired HNSCC and normal tissues. We found that twelve miRNAs are downregulated and six are upregulated in HSCC tissues (Table 1). Among these, levels of miR-141 were significantly reduced in HNSCC tissues. Further analysis revealed that levels of miR-141 expression are lower in HNSCC tissues than adjacent normal tissues and that the decrease correlated with the occurrence and development of HNSCC (Table 2, Fig. 1A, B).

**Table 1:** Differentially expressed miRNAs in HNSCCs.

miRNA	Fold change (HNSCC/adjacent tissues)	Trend
miR-106b-5p	31.37	Down
miR-141-3p	63.25	Down
miR-142-3p	11.42	Down
miR-149-3p	13.25	Down
miR-150-5p	27.37	Up
miR-191-5p	9.62	Up
miR-195-5p	12.10	Down
miR-21-5p	25.62	Up
miR-28-3p	14.12	Down
miR-30b-3p	39.42	Down
miR-3689c	9.64	Up
miR-425-5p	8.12	Down
miR-4728-5p	10.04	Down
miR-4779	12.94	Up
miR-541-3p	34.72	Down
miR-548g-3p	22.73	Up
miR-574-3p	39.64	Down
miR-590-5p	41.37	Down

**Table 2:** Relationship between miR-141 and HNSCCs.

Variables	Description	No. of patient	miR-141 expression		^÷2^	*P*
			Low High			
Gender	Male	18	10	8	0.13	0.71
	Female	12	9	3		
Age(years)	<40	14	8	6	0.43	0.51
	≥40	16	11	5		
Family history	No Yes	24 6	13 6	11 0	4.34	0.04
TNM grade	IIA	14	5	9	8.87	0.01
	IIB III	13 3	11 3	2 0		

### EGFR is a direct target of miR-141

**Figure 2:**
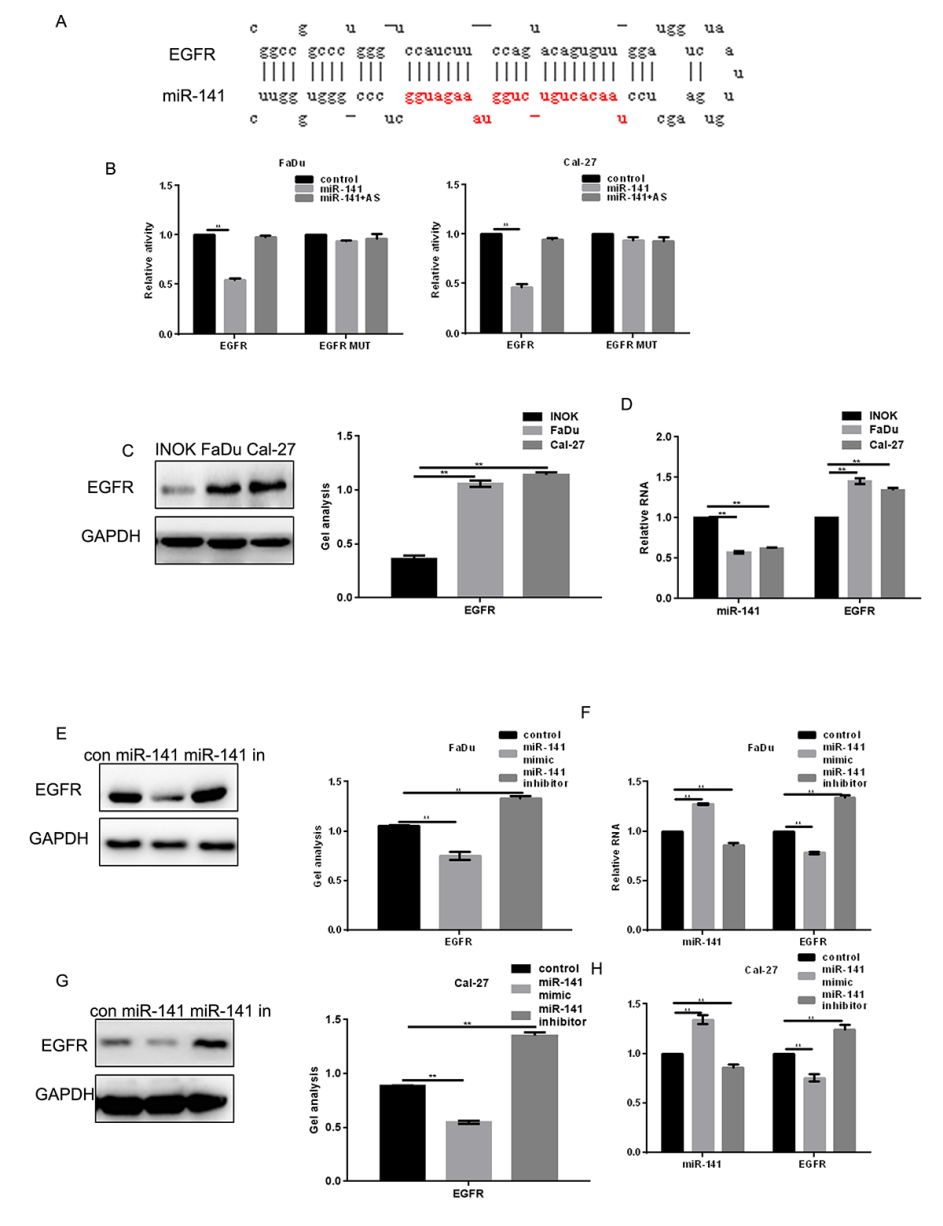
**EGFR is a direct target of miR-141.** (**A**) Prediction of miR-141 binding sites in the EGFR 3’-UTR. (**B**) Interaction between miR-141 and the EGFR 3’-UTR in FaDu and Cal-27 cells tested in luciferase assays. AS, antisense. (**C**) Western blots showing expression of EGFR in INOK, FaDu and Cal-27 cells. (**D**) Western blots showing expression of miR-141 and EGFR in INOK, FaDu and Cal-27 cells. (**E, F**) Western blot and real-time PCR showing co-expression of EGFR in FaDu cells with miR-141 mimic or inhibitor. (**G, H**) Western blot and real-time PCR showing co-expression of EGFR in Cal-27 with miR-141 mimic or inhibitor. Bars depict the mean ± SD. ** P< 0.05 vs. INOK cells (C, D) or control (E, F).

EGFR is highly expressed in HNSCC tissues (Fig. 1C). Moreover, patients showing higher EGFR expression had shorter survival times than those with lower EGFR expression (Fig. 1D). Levels of miR-141 expression correlated negatively with EGFR expression in HNSCC (Fig. 1E). MiRDB analysis showed that miR-141 bound the 3’-UTR of EGFR via (Fig. 2A). Luciferase assays showed that when FaDu and Cal-27 cells were cotransfected with EGFR and miR-141, luciferase activity was suppressed. By contrast, no suppression was detected when EGFR-mut or miR-141 antisense was transfected into the cells (Fig. 2B). Consistent with the luciferase activity, we observed that EGFR mRNA and protein expression was relatively high, while miR-141 expression was relatively low, in HNSCC cells (Fig. 2C, D). In addition, expression of miR-141 mimic in these cells decreased expression of EGFR, while an miR-141 inhibitor enhanced EGFR expression (Fig. 2E-H).

### miR-141 inhibits HNSCC cell proliferation

**Figure 3:**
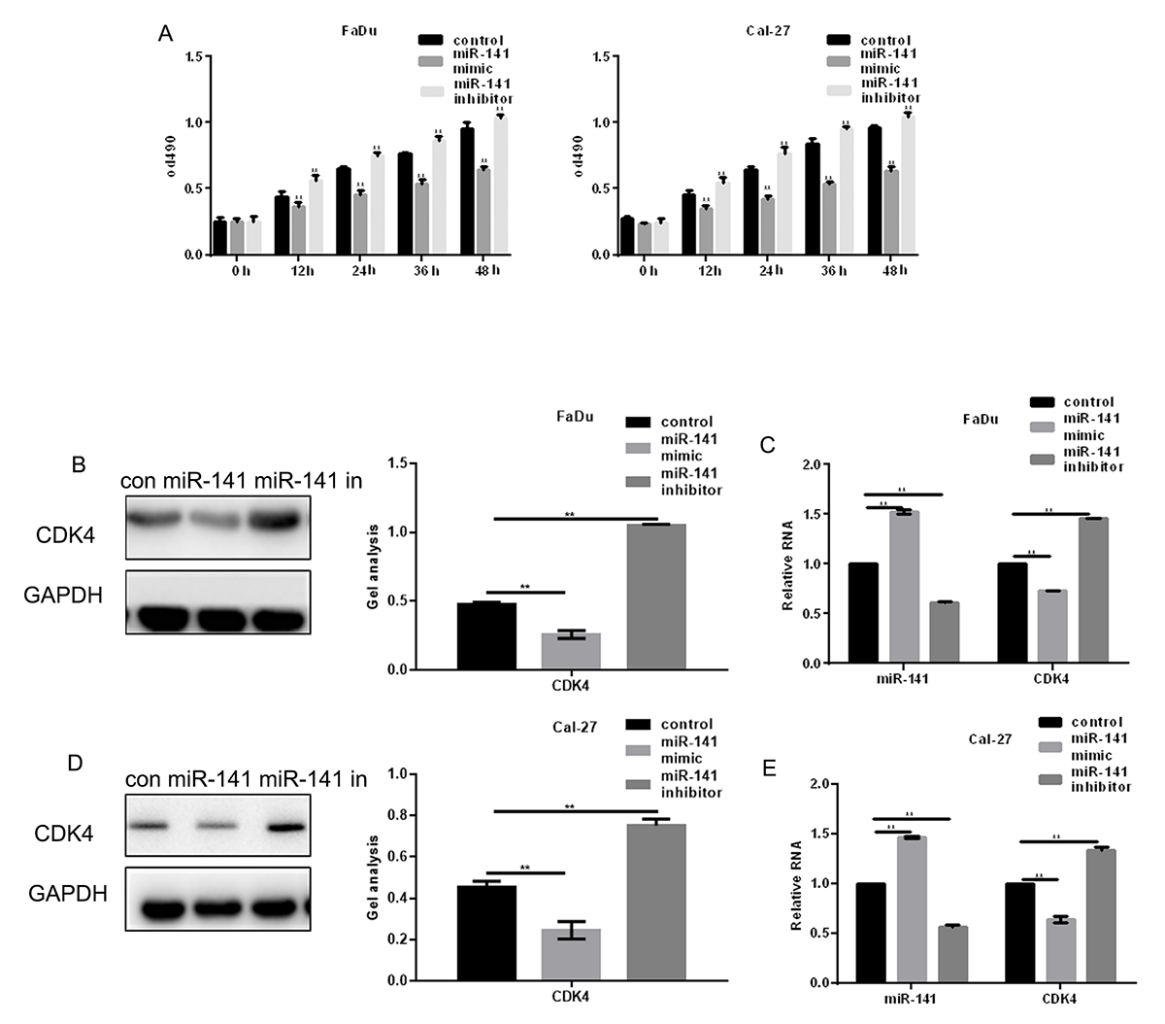
**MiR-141 inhibits HNSCC cell proliferation.** (**A**) MTT assays showing the effect of miR-141 mimic and inhibitor on FaDu and Cal-27 cell proliferation. (**B-E**) Western blots and real-time PCR were used to detect the expression of CDK4 in FaDu and Cal-27 cells expressing miR-141 mimic or inhibitor. Bars depict the mean ± SD. ** P< 0.05 vs. control.

MTT assays showed that an miR-141 mimic inhibited proliferation of HNSCC cells, while an miR-141 inhibitor promoted their proliferation (Fig. 3A). EGFR is known to increase cell proliferation by enhancing expression of CDK4 [8]. We found that miR-141 mimic suppressed CDK4 expression, while an miR-141 inhibitor had the opposite effect (Fig. 3B-E).

### miR-141 promotes HNSCC apoptosis

**Figure 4:**
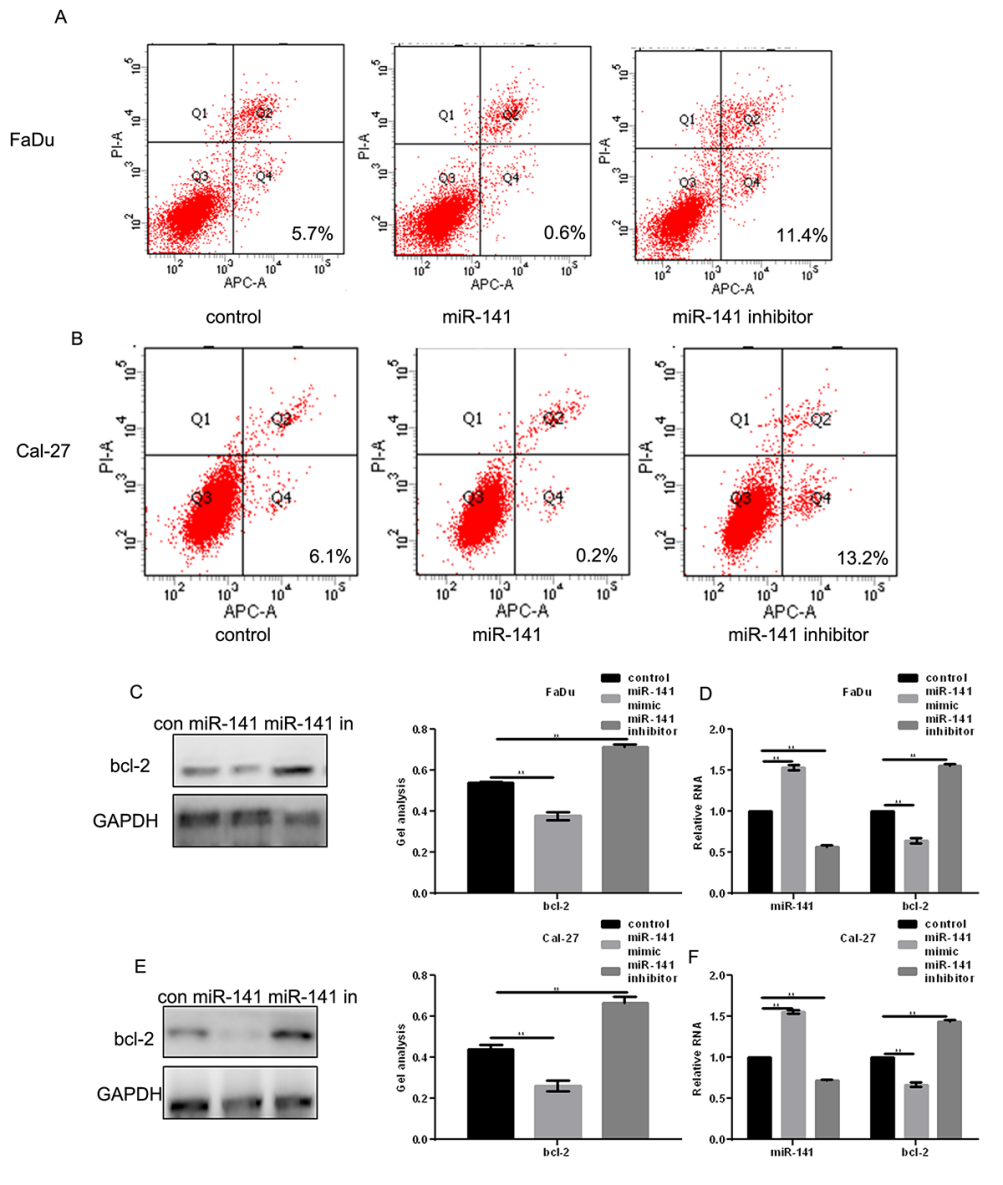
**MiR-141 promotes HNSCC apoptosis.** (**A, B**) Flow cytometry AV-PI assays showing the effect of miR-141 on FaDu and Cal-27 cell apoptosis. (**C-F**) Western blots and real-time PCR showing the effect of miR-141 mimic and inhibitor on bcl-2 expression in FaDu and Cal-27 cells. Bars depict the mean ± SD. ** P< 0.05 vs. control.

AV-PI assays showed that transfection of miR-141 mimic promoted apoptosis of FaDu and Cal-27 cells as compared to control (Fig. 4A, B). Western blotting and real-time PCR showed that this may reflect an inhibitory effect of miR-141 on expression of bcl-2 (Fig. 4C-F), a downstream EGFR effector [9].

### miR-141 inhibits HNSCC metastasis

**Figure 5:**
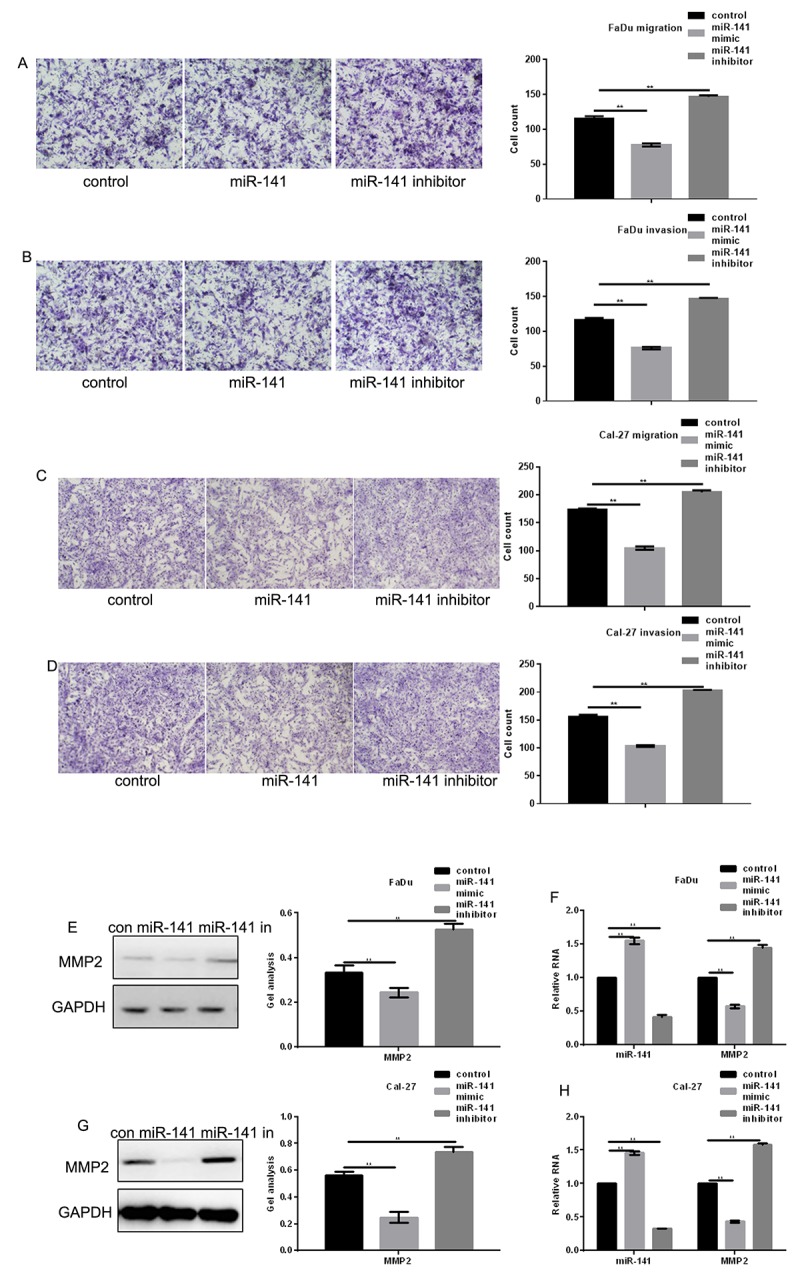
**MiR-141 inhibits HNSCC metastasis.** (**A-D**) Transwell assays with and without Matrigel showing the effect of miR-141 on FaDu and Cal-27 cell migration and invasion. Left panels: representative images of migrated cells under the indicated conditions. Right panels: Group data showing counts of migrated cells. Bars depict the mean ± SD of three experiments. (**E-H**) Western blots and real-time PCR the effect of miR-141 mimic and inhibitor on MMP2 expression in FaDu and Cal-27 cells. Bars depict the mean ± SD. ** P< 0.05 vs. control.

Transwell assays were used to assess the effect of miR-141 on FaDu and Cal-27 cell migration. The results indicated that miR-141 overexpression reduced the number of migrating cells, and miR-141 inhibition had the opposite effect (Fig. 5A-D). Western blot and real-time PCR showed that matrix metalloproteinase 2 (MMP2), an enzyme involved in breaking down extracellular matrix and known to be positively regulated by EGFR [10], was significantly downregulated by miR-141 (Fig. 5E-H).

### miR-141 inhibits FaDu cell invasion *in vivo*

**Figure 6:**
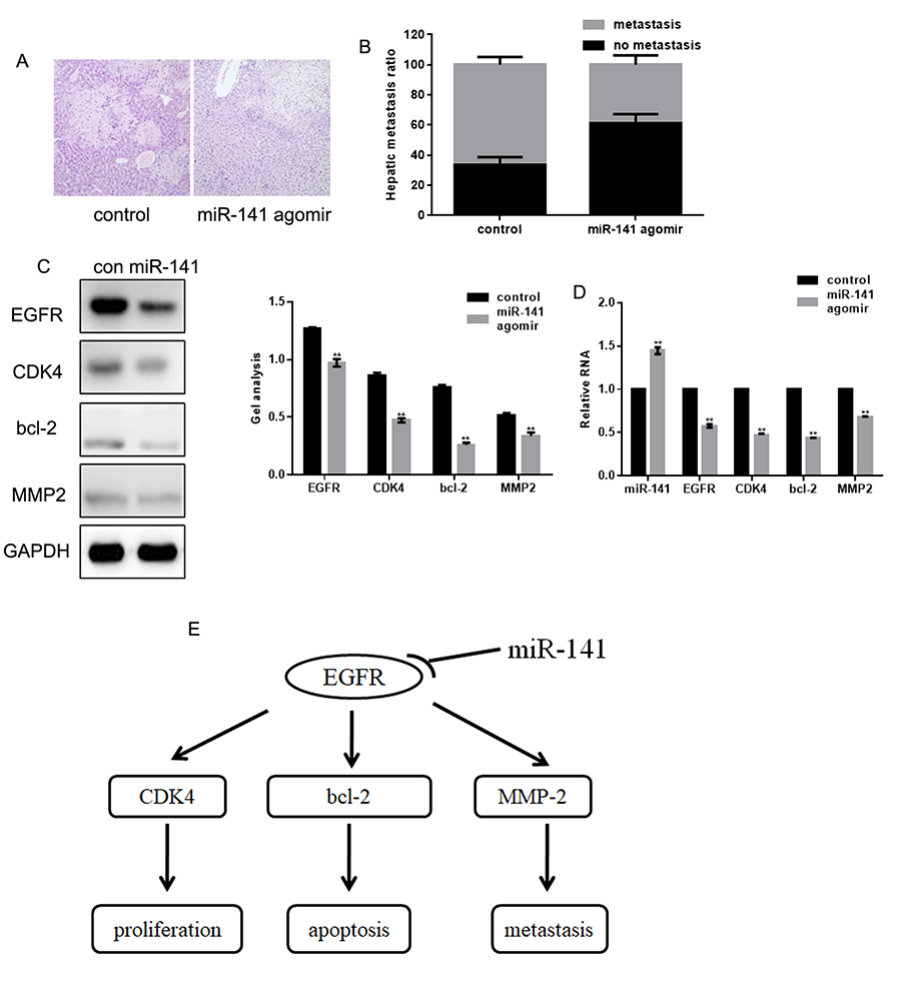
**MiR-141 inhibits FaDu cell invasion *in vivo.*** (**A**) Livers were dissected and stained with hematoxylin and eosin. (**B**) Bar graph showing the effect of miR-141 on the relative levels of hepatic metastasis. (**C, D**) Western blots and real-time PCR showing the effect of miR-141 on expression of EGFR, CDK4, bcl-2 and MMP2 in tissues. Bars depict the mean ± SD. ** P< 0.05 vs. control.

FaDu cells stably expressing control or miR-141 agomir were injected into BALB/c nude mice via the tail vein to examine the effect of miR-141 on cell invasiveness *in vivo*. Hematoxylin-eosin staining of liver tissues showed that miR-141 inhibited the hepatic metastatic activity of FaDu cells (Fig 6A, B). We also found that overexpression of miR-141 can downregulate the expression of EGFR, CDK4, bcl-2 and MMP2 *in vivo* (Fig 6C, D). These findings suggest that by suppressing EGFR, miR-141 inhibits the growth and metastasis of HNSCC (Fig 6E).

## DISCUSSION

In many tumors, levels of miR-141 expression are lower than in healthy tissue, and its increased expression can inhibit tumor progression [11]. For example, miR-141 has been shown to inhibit the development of gastric, prostate, colorectal and liver cancers by inhibiting cell proliferation and metastasis and promoting cell apoptosis [12–14]. In our study, we found that miR-141 was weakly expressed in HNSCC tissues and that higher expression of miR-141 improves patients’ survival time.

EGFR plays important roles in angiogenesis and in the migration, proliferation and apoptosis of tumor cells [15]. In the present study, we demonstrated that EGFR is highly expressed in HNSCC tissues and that miR-141 targeted and bound to EGFR. As a result, overexpression of miR-141 can inhibit the EGFR expression at both the protein and mRNA level.

We also found that overexpression of miR-141 inhibits HNSCC cell proliferation by inhibiting the CDK4 expression and promotes HNSCC cell apoptosis by inhibiting bcl-2. In addition, miR-141 inhibited HNSCC cell migration and invasion by inhibiting expression of MMP2. Because CDK4, bcl-2, and MMP2 are all regulated by EGFR [16,17] and do not bind miR-141 (predicted by miRDB), we suggest the inhibitory effect of miRNA-141 on the HNSCC cell growth and metastasis reflects, at least in part, its targeting of EGFR. We also demonstrated that miR-141 agomir inhibits HNSCC metastasis *in vivo* and that miR-141 also inhibits expression of EGFR, CDK4, bcl-2 and MMP2 *in vivo*.

In summary, our results suggest that miR-141 functions as a tumor suppressor in HNSCC and that it suppresses tumor growth and metastasis by suppressing EGFR signaling. MiR-141 thus appears to be a potentially useful therapeutic target in the treatment of HNSCC.

## Conclusion

MiR-141 functions as a tumor suppressor in HNSCC and that it suppresses tumor growth and metastasis by suppressing EGFR signaling.

## MATERIALS AND METHODS

### Samples

Thirty samples of fresh primary HNSCC and the adjacent normal epithelial tissues were obtained during surgery at Shengjing Hospital of China Medical University between 2011-2017. These included tongue SCC (56.67%, 17), oropharynx SCC (33.33%, 10), mouth floor SCC (6.67%, 2) and buccal mucosa SCC (3.33%, 1). None of the patients were treated with chemotherapy before the operation. The average age of the patients was 42.78 years (range: 29 to 63 years), and 18 of the patients were male. This study was approved by the Research Ethics Committee of Shengjing Hospital of China Medical University, and all patients provided written informed consent.

### MicroRNA microarray analysis

Total RNA was extracted from tissues using a miRNA isolation kit (Takara, Japan) according to the manufacturer’s protocol. MicroRNA microarray analysis was carried out by Huibai Biotechnology (Shenyang, China). Differentially expressed miRNAs were identified using a Moderated t-test, with the Benjamini-Hochberg correction (adjusted P<0.05 and fold change >2).

### Cell lines and cell cultures

The FaDu (human pharynx SCC) and Cal-27 (human tongue SCC) HNSCC cell lines and immortalized normal oral keratinocytes (INOKs) were purchased from the cell bank of the Chinese Academy of Sciences (Beijing, China). Cells were cultured in DMEM medium (Invitrogen, Beijing, China) supplemented with 10% fetal bovine serum (FBS) (Gibco, Beijing, China) at 37°C in an atmosphere of 95% air/5% carbon dioxide.

### Real-time PCR

Total RNA was extracted from tissues and cells using a Trizol kit (Takara, Japan), after which cDNA was obtained using a TaqMan® reverse transcription kit (Ribobio, China). The real-time PCR protocol entailed incubation at 95°C for 10 min, followed by 40 cycles at 95°C for 15 s and 60°C for 1 min. U6 and GAPDH were used as internal controls. A stem-loop RT-PCR assay was used to detect expression of miRNAs, as described previously [18,19]. Primer sequences are listed in Table 3.

**Table 3:** Primer sequences.

Name	Forward primer (5'->3')	Reverse primer (5'->3')
miR-141	ACACTCAGGTAGAAATG	ATTGTGACACCGAGTAC
U6	CTCGCTTCGGCAGCACA	ACGCTTCACGAATTTGC
h-EGFR	GACAGGCCACCTCGTCG	TGCGTGAGCTTGTTACTCGT
h-CDK4	CACTCTAACACTGTCTGG	ACATCGTTACCGAGAGTA
h-bcl-2	TTCTTTGAGTTCGGTGGGGTC	TGCATATTTGTTTGGGGCAGG
h-MMP2	CGCATCTGGGGCTTTAAACAT	CCATTAGCGCCTCCATCGTA
h-GAPDH	GACAGTCAGCCGCATCTTCT	GCGCCCAATACGACCAAATC
m-EGFR	GGCTATCTGACAGAGGTCGC	CTCTCACGGATTACCGCTCC
m-CDK4	TGATGCGCCAGTTTCTAAGAGG	GGTCGGCTTCAGAGTTTC
m-Bcl-2	TGCGTGAAGGCTTGAGATGT	TCCCCCTTTCCTAGACCCAG
m-MMP2	GCACACCAGGTGAAGGATGT	AAACGAGCGAAGGGCATACA
m-GAPDH	CAGGTTGTCTCCTGCGACTT	TATGGGGGTCTGGGATGGAA

All the reactions were carried out as described previously [20].

### Western blotting

Total proteins were isolated from the cells using RIPA buffer. Thirty-microgram aliquots of protein were boiled for 5 min, separated by sodium dodecyl sulfate-polyacrylamide gel electrophoresis, and transferred to polyvinylidene fluoride membranes (Bio-Rad Laboratories, Tianjin, China). After incubation in blocking solution for 2 h at room temperature, the membranes were incubated with primary antibodies overnight at 4°C. Membranes were probed with anti-EGFR (1:500), anti-CDK4 (1:500), anti-bcl-2 (1:500), anti-MMP2 (1:500), anti-GAPDH (1:1000), HRP-conjugated anti-mouse (1:5000) and anti-rabbit (1:5000) (Santa Cruz Biotechnology, Texas, USA). GAPDH was used as a loading control.

### Luciferase assays

The EGFR and EGFR-mut (the miR-141 binding site was mutated) 3’-UTR were cloned into the pMIR-REPORTTM vector (Ambion, China). For reporter assays, 1×10^4^ cells were plated in a 96-well plate. After 24 h, the cells were cotransfected with miR-141 mimic, antisense or control (Ribobio, Guangzhou, China) plus 100 ng of EGFR or EGFR-MUT using Higene. Lysates were collected 48 h after transfection, and luciferase activity was assessed using a Dual Luciferase Reporter Assay System (Promega, USA).

### MTT assays

Cells were plated into a 96-well plate at a density of 1,000 cells per well. After 24 h, the cells were transfected with the miR-141 mimic, miR-141 inhibitor or control. Cell proliferation was analyzed after 0, 12, 24 and 48 h using MTT assays (Beyotime Biotechnology, Tianjin, China) according to the manufacturer’s protocol, measuring absorbance at 490 nm. All assays were performed in triplicate wells, and experiments were independently performed at least three times.

### Transwell assays

After cells were transfected with the miR-141 mimic, miR-141 inhibitor or control, 1×10^5^ cells in serum-free medium were seeded to the upper chamber of Transwell plates left bare or coated with Matrigel. After 8 h, cells on the lower side of the chamber were fixed and stained with 0.4% trypan blue (Beyotime Biotechnology, Tianjin, China) and counted under a microscope.

### Annexin V-FITC/PI (propidium iodide) assays

Cells were seeded into 6-well plates to a density of 8,000 cells/well and incubated for 24 h. The cells were then transfected with miR-141 mimic, miR-141 inhibitor or control. After an additional 24 h, cellular apoptosis was analyzed using an AV-PI assay (Beyotime Biotechnology, Tianjin, China) according with the manufacturer’s protocol. Suspended cells were incubated with AV-PI for 5 min at room temperature. Apoptosis was then assessed using flow cytometry in at least three independent experiments.

### Nude mouse experiment

FaDu cells infected with control or miR-141 agomir were used for *in vivo* liver metastasis assays. Five- to six-week-old female, athymic nude BALB/c mice (animal experiment center, Wuhan University, China) were injected via the tail vein with 1×10^6^ cells suspended in 1 ml of saline. Six weeks after the injection, liver samples were collected for histological examination. All procedures were conducted in accordance with the Guide for the Care and Use of Laboratory Animals (NIH publication no. 80-23, revised 1996) and the institutional ethical guidelines of Shengjing Hospital of China Medical University.

### Histopathology

Liver specimens were fixed with 4% paraformaldehyde and cut into serial sections (2 μm). The sections were then deparaffinized in xylene, rehydrated through a descending graded ethanol series, and stained with hematoxylin and eosin. The stained sections were dehydrated through an ascending graded ethanol series and mounted on slides. The pathological changes were observed under a light microscope.

### Bioinformatic and statistical analyses

MiRDB (http://www.mirdb.org/) was used to predict the target genes of miR-141. All statistical analyses were performed using SPSS 13.0 software (SPSS Inc., Chicago, IL, USA). All data are presented as the mean ± standard deviation. Groups were compared using two tailed Student's t-test and one-way ANOVA. Values of P < 0.05 were considered statistically significant.

### Ethics approval and consent to participate

Research involving human subjects, human material, or human data, was performed in accordance with the Declaration of Helsinki and was approved by the Research Ethics Committee of Shengjing Hospital of China Medical University (R20141121).

### Consent for publication

Written informed consent for the publication of all manuscript details was obtained from Shengjing Hospital of China Medical University.
